# Acute Presentation of Simultaneous Liver Abscesses Caused by Streptococcus constellatus

**DOI:** 10.7759/cureus.8497

**Published:** 2020-06-07

**Authors:** Muhammad Faisal Riaz, Alvaro Genao, Ibrahim Omore

**Affiliations:** 1 Internal Medicine, Harlem Hospital Center, New York, USA; 2 Internal Medicine: Gastroenterology, Columbia University School of Physicians and Surgeons at Harlem Hospital Center, New York, USA; 3 Internal Medicine, Columbia University College of Physicians and Surgeons at Harlem Hospital Center, New York, USA

**Keywords:** liver abscess, streptococcus constellatus, drainage

## Abstract

Simultaneous liver abscesses are rarely seen and reported. We are reporting a case of two simultaneous, complex liver abscesses in a patient who had no evidence of liver abscess on cross-sectional imaging close to three months prior to this presentation. These abscesses were 7-8 cm in size, large, and septated. Microbiological studies were positive for Streptococcus constellatus, which is a known cause of pyogenic liver abscess. In our patient, pyogenic liver abscesses were associated with bacteremia and sepsis. This patient was managed with broad spectrum parenteral antibiotics and percutaneous drainage with improvement in clinical condition. This patient was discharged home with a peripherally inserted central catheter (PICC) line in place to complete a six-week course of parenteral antibiotics. A complete history and physical with pertinent examination findings are key to diagnosis of liver abscess. S. constellatus should be considered in the differential diagnosis of patients with liver abscess and sepsis.

## Introduction

Pyogenic liver abscesses in the majority of cases are caused by *Escherichia coli* or *Klebsiella pneumoniae*. *Streptococcus constellatus* is a rare, but known cause of pyogenic liver abscess. This bacterium is Gram-positive, part of the normal flora of the oro-gastrointestinal and genitourinary tracts, and can lead to bacteremia and sepsis. We present a rare case of two, simultaneous liver abscesses in a 41-year-old male who came to the ER with severe right upper quadrant (RUQ) pain and fever. He responded well to parenteral antibiotics and percutaneous drainage. Liver abscesses can be caused by hematogenous seeding or from local spread through hepatobiliary system. Our case is unique and rare in its presentation in that this patient presented with two large liver abscesses and sepsis while he had no antecedent history of RUQ pain or liver pathology in his last hospital presentation three months prior.

## Case presentation

Here we report a case of a 41-year-old man with past medical history (PMH) of asthma, urinary tract infection (UTI), erectile dysfunction, and diverticulosis with a prior episode of diverticulitis who had presented with left lower quadrant (LLQ) pain and managed for complicated UTI secondary to prostatitis/nephrolithiasis in July 2019, three months prior to the current presentation. Blood and urine cultures did not reveal any growth at that time and CT of abdomen and pelvis demonstrated diverticulitis of descending colon, thickening of bladder wall, and prostatic calcifications, with no liver lesions. He was discharged home on oral levofloxacin and his pain resolved with completion of outpatient course of oral antibiotics.

He came to the ER three months later in October 2019 with a two-day history of RUQ pain, 9/10 in intensity, not related to oral intake, and relieved by lying supine. It was associated with anorexia and malaise. He also mentioned a one-week history of fever, rigors, and generalized weakness. Review of systems were remarkable for foul-smelling urine with associated, intermittent LLQ pain. He reported nausea without vomiting. Of note, he denied recent travel and change in dietary habits. He has not been sexually active for the past year and he had an index colonoscopy in 2004.

On exam in the ER, his BP was 121/79, pulse 111, temp. 101.7°F, respiratory rate (RR) 19, saturating at 95% on room air. His mucous membranes were dry, abdomen was tender in RUQ. Chest was clear and rectal exam was negative for mass or active bleeding with a normal sphincter tone. His labs were significant for white blood cell (WBC) of 17.7 with left shift (neutrophil of 85.7 and bands 25%), hemoglobin (Hb) of 12.8, potassium of 3.3, magnesium of 1.5 and lactate of 2.2, blood urea nitrogen (BUN)/creatinine of 21/1.0, alkaline phosphatase of 136, aspartate aminotransferase (AST) 218, alanine transaminase (ALT) 242 and international normalized ratio (INR) of 1.3. Urine analysis (UA) was positive for nitrites, LE 2+, packed field with WBC and many bacteria on urine microscopy.

**Figure 1:**
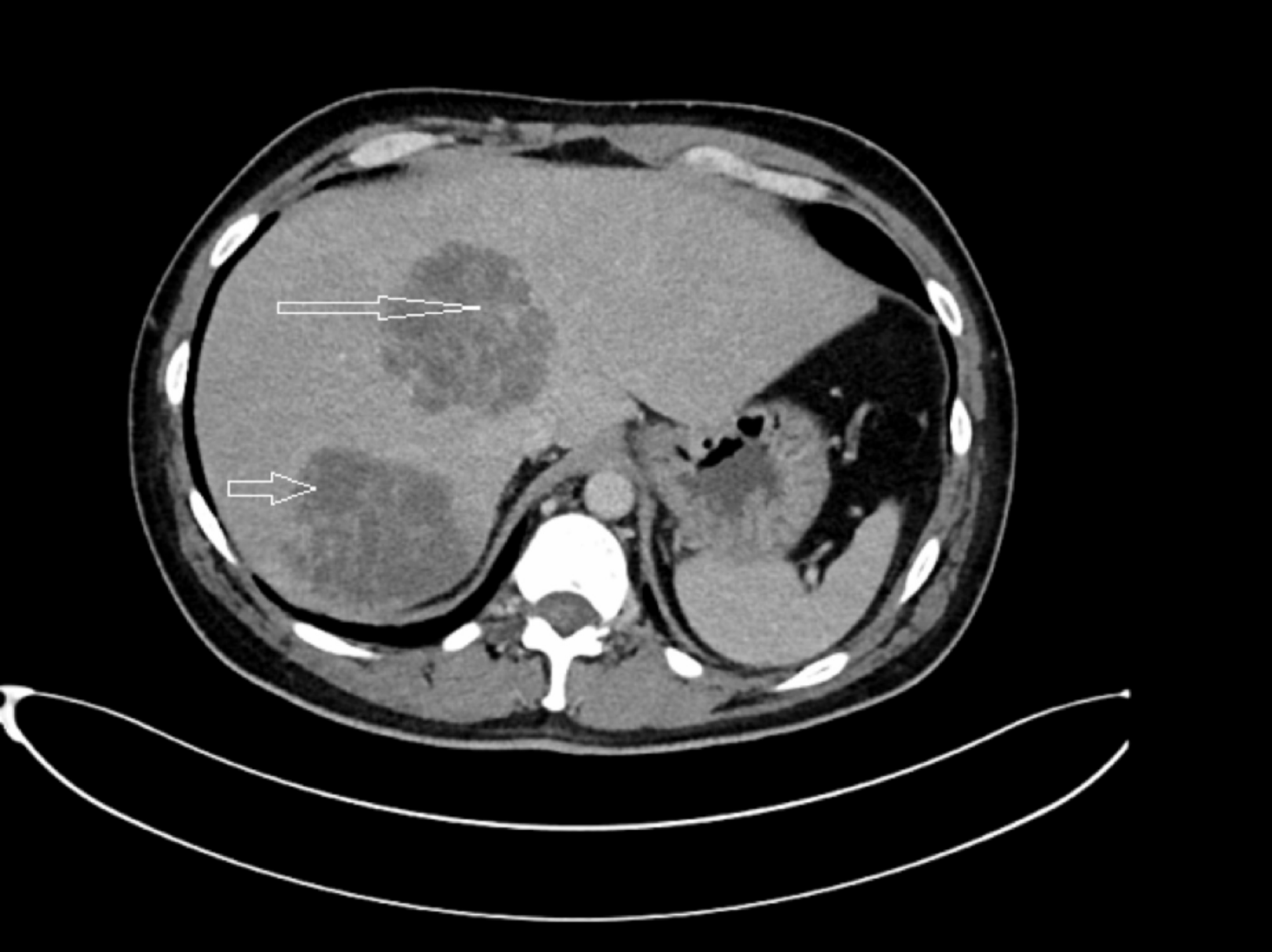
CT abdomen done in October 2019. Depicting two large, multiloculated abscesses in right lobe of liver, measuring each 7-8 cm in size.

**Figure 2:**
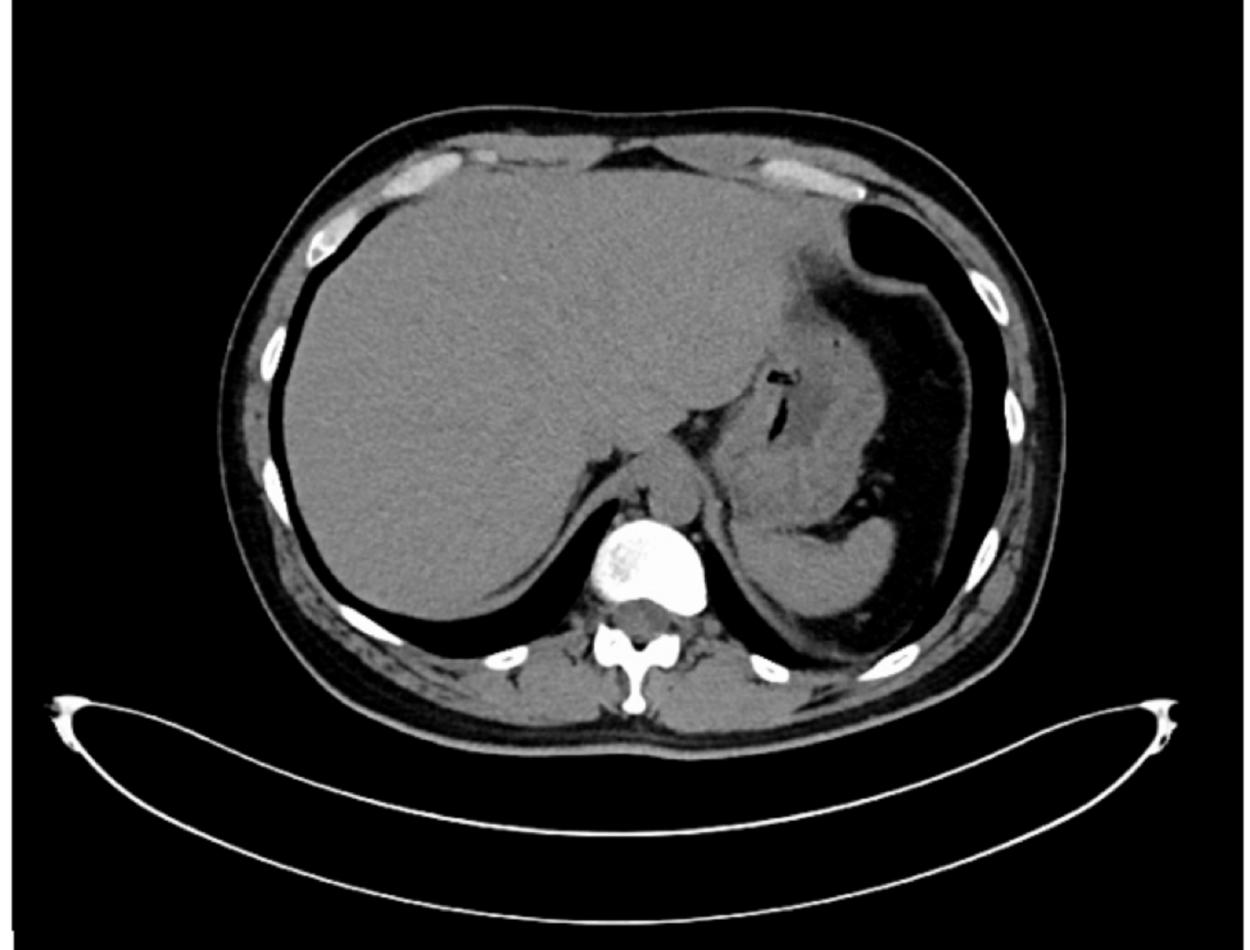
CT abdomen done in July 2019. Depicting no liver mass or abscess seen.

Chest X-ray (CXR) did not demonstrate any acute cardiopulmonary disease. CT abdomen and pelvis revealed two multilocular, hypoattenuating lesions in the right lobe of the liver which were 7-8 cm in size (Figure 1), new as compared to previous CT scan done three months prior (Figure 2). Cross-sectional imaging was also suggestive of sigmoid diverticulitis and colo-vesical fistula. At this point, the main focus was on multifocal, metastatic infection to the liver versus primary liver abscesses. The patient was admitted to the ICU for intensive monitoring and management of sepsis secondary to hepatic abscesses/complicated UTI. Blood and urine cultures were taken before the initiation of broad-spectrum, intravenous antibiotics.

He underwent percutaneous drainage of both liver collections and fluid analysis was performed. The fluid was tested for ova and parasite with negative results. Cytology was negative for malignant cells. Fluid microscopy revealed abundant neutrophils consistent with abscess. Cultures were positive for *S. constellatus*. Blood cultures isolated *S. constellatus* which was concordant with fluid studies. Urine culture was significant for *E. coli*. Serological testing for Echinococcus antibody was negative. Based on the culture and sensitivity of liver fluid collections, he was started on levofloxacin and metronidazole. The patient was managed for Gram-positive bacteremia and repeat cultures after initiation of parenteral antibiotics were negative. A peripherally inserted central catheter (PICC) line was placed to continue intravenous antibiotics for six weeks.

**Figure 3:**
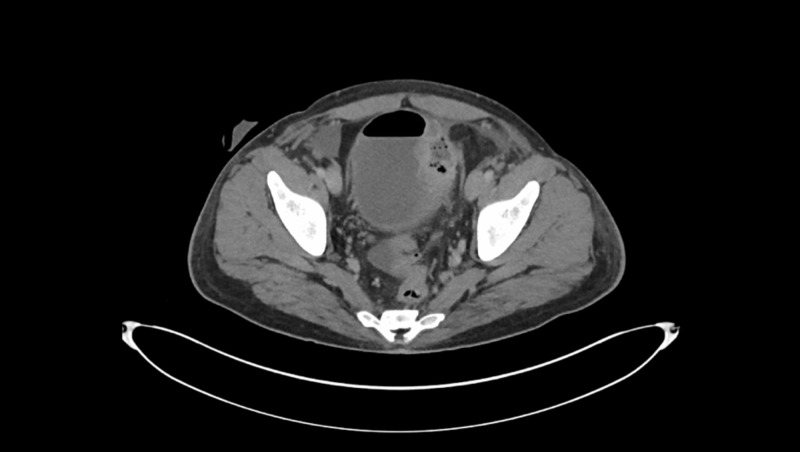
CT abdomen/pelvis done in October 2019. Depicting left pelvic collection possible sigmoid abscess/communication of colovesical fistula.

Follow-up CT revealed a sigmoid abscess and findings consistent with colo-vesical fistula (Figure 3). The sigmoid abscess was drained percutaneously, but not cultured. A diverting loop ileostomy was performed for initial management of colo-vesical fistula with plans for definitive surgery in the future. Work up for inflammatory bowel disease was negative including anti-Saccharomyces cerevisiae antibodies (ASCA) and anti-neutrophilic cytoplasmic autoantibody (ANCA). HIV and Hepatitis B and C serologies were negative.

**Figure 4:**
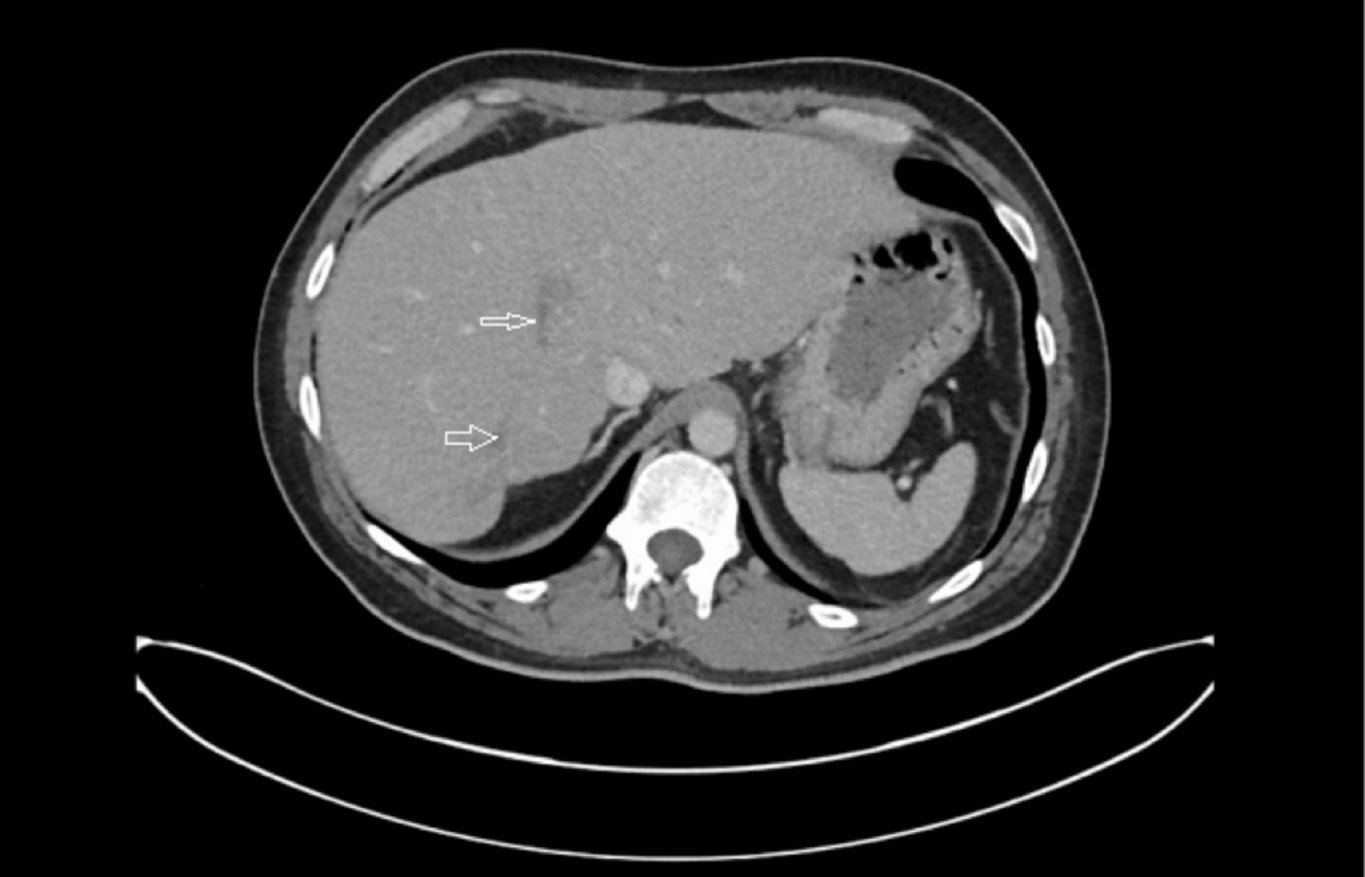
CT abdomen done in December 2019. Depicting resolution of both liver abscesses after percutaneous drainage and six weeks of IV antibiotics.

He was discharged home with PICC line in place with arrangements for outpatient IV antibiotics. During outpatient follow up, both liver abscess were fully resolved (Figure 4). He completed six weeks of IV antibiotics and the PICC line was removed. He will follow up with urology for definitive repair of colovesical fistula.

## Discussion

Liver abscesses make up a significant portion of all visceral abscesses. Diverticulitis is one of the most recognized causes of liver abscess [1]. Liver abscesses may arise from hematogenous spread or local spread from contiguous sites of infection within the peritoneal cavity [1]. Risk factors include diabetes mellitus, hepatobiliary and pancreatic disease, colon cancer, portal vein pyemia, and liver transplant [2]. Occasionally, abscesses arise from surgical or penetrating wounds and ingested foreign body [3]. In majority of cases, pyogenic liver abscesses are caused by *K. pneumoniae* and *E. coli*, but *S. constellatus* (member of *S. melleri* group) is also a known cause of liver abscess [1, 4-5]. A monomicrobial liver abscess due to a streptococcal or staphylococcal species should prompt evaluation for an additional source of infection, in particular infectious endocarditis and abscesses in other organs. *S. constellatus* has been increasingly reported in abscess aspirate and blood cultures in recent years [6]. A case study of 73 patients showed four cases of uncommon streptococcus species causing liver abscess [7].

Fever, chills, and RUQ abdominal pain are the most common presenting signs of liver abscess, seen in a majority of cases [7]. Nonspecific symptoms such as anorexia, weight loss, nausea, and vomiting may also develop. Many patients develop RUQ tenderness, hepatomegaly, and jaundice. Diagnostic studies of the abdomen, especially the RUQ, should be a part of any workup for fever, abdominal pain, and RUQ tenderness. Sepsis in the setting of liver abscess warrants aggressive management and may lead to increased mortality [8-9].

Imaging studies are the most reliable method for diagnosing liver abscesses including ultrasonography, CT, indium-labeled WBC or gallium scan, and MRI. CT or ultrasound-guided drainage of all suspected liver abscesses is essential to confirm the diagnosis and to identify the microbial pathogens involved. In cases of small, single abscesses, needle aspiration may be sufficient for therapeutic abscess drainage; in other cases, a drainage catheter may be warranted [1, 5].

Empiric, broad-spectrum, parenteral antibiotics should be administered pending aspiration of the abscess and microbiological analysis of abscess contents and blood. Mohanty et al. had reported a case of liver abscess due to *S. constellatus* managed with surgical intervention and parenteral IV antibiotics with clinical recovery and abscess resolution [5]. Liver abscesses with *S. constellatus* may lead to rupture and need surgical drainage with laparotomy [4].

## Conclusions

Pyogenic liver abscesses should be considered strongly in the differential diagnosis of patients presenting with fever, RUQ pain, and chills. Blood cultures should be drawn before the initiation of broad-spectrum antibiotics. *S. constellatus* is a rare, but well-known cause of pyogenic liver abscess. Antibiotic coverage should be focused and narrowed based on the results of culture and sensitivity. Percutaneous drainage and six weeks of IV antibiotic coverage provide symptomatic relief, source-directed therapy, and resolution of liver abscesses. Repeat interval imaging is also needed to confirm resolution.
